# Gut-Liver Immune Traffic: Deciphering Immune-Pathogenesis to Underpin Translational Therapy

**DOI:** 10.3389/fimmu.2021.711217

**Published:** 2021-08-25

**Authors:** Amber G. Bozward, Vincenzo Ronca, Daniel Osei-Bordom, Ye Htun Oo

**Affiliations:** ^1^Centre for Liver and Gastrointestinal Research and National Institute for Health Research (NIHR) Birmingham Biomedical Research Centre, Institute of Immunology and Immunotherapy, University of Birmingham, Birmingham, United Kingdom; ^2^Liver Transplant and Hepatobiliary Unit, Queen Elizabeth Hospital, University Hospital of Birmingham NHS Foundation Trust, Birmingham, United Kingdom; ^3^Centre for Rare Diseases, European Reference Network - Rare Liver Centre, Birmingham, United Kingdom; ^4^Birmingham Advanced Cellular Therapy Facility, University of Birmingham, Birmingham, United Kingdom; ^5^Queen Elizabeth Hospital, University Hospital of Birmingham National Health Service (NHS) Foundation Trust, Birmingham, United Kingdom

**Keywords:** plasticity, therapy, liver disease, Gut microbiota, Metabolites, PSC, PBC, IBD

## Abstract

The tight relationship between the gut and liver on embryological, anatomical and physiological levels inspired the concept of a gut-liver axis as a central element in the pathogenesis of gut-liver axis diseases. This axis refers to the reciprocal regulation between these two organs causing an integrated system of immune homeostasis or tolerance breakdown guided by the microbiota, the diet, genetic background, and environmental factors. Continuous exposure of gut microbiome, various hormones, drugs and toxins, or metabolites from the diet through the portal vein adapt the liver to maintain its tolerogenic state. This is orchestrated by the combined effort of immune cells network: behaving as a sinusoidal and biliary firewall, along with a regulatory network of immune cells including, regulatory T cells and tolerogenic dendritic cells (DC). In addition, downregulation of costimulatory molecules on hepatic sinusoids, hepatocytes and biliary epithelial cells as well as regulating the bile acids chain also play a part in hepatic immune homeostasis. Recent evidence also demonstrated the link between changes in the gut microbiome and liver resident immune cells in the progression of cirrhosis and the tight correlation among primary sclerosing cholangitis (PSC) and also checkpoint induced liver and gut injury. In this review, we will summarize the most recent evidence of the bidirectional relationship among the gut and the liver and how it contributes to liver disease, focusing mainly on PSC and checkpoint induced hepatitis and colitis. We will also focus on completed therapeutic options and on potential targets for future treatment linking with immunology and describe the future direction of this research, taking advantage of modern technologies.

## Introduction

The strategic anatomical localization and blood supply from the portal vein links the liver and the gut tightly both anatomically and functionally. A wide variety of commensal microorganisms, microbial and food antigens, or xenobiotics, reach the liver through the portal blood from the spleen and mesenteric veins, exposing the liver to continuous immunogenic stimuli. As a frontline organ facing the influx from gut mucosal barriers, the liver continuously maintains the balance between immunity and tolerance to maintain homeostatic state of human body.

On the other hand, molecules secreted by the liver in the bile reach the gut, to configure a bidirectional crosstalk the so-called gut-liver axis. Molecules that make up the bile salts can shape the gut immune system and regulate the gut microflora (1–3).

### Gut Microbiota

The gut microbiome has recently been found to be an active part in the complex relationship between the liver and the gut (4). It is composed of a wide collection of microorganisms, and the dominant phyla are Firmicutes, Actinobacteria, Bacteroidetes, Proteobacteria, Fusobacteria, and Verrucomicrobia, with the Firmicutes and Bacteroidetes representing over 90% of the flora (5). The composition of the gut microflora changes during an individual’s lifespan (6, 7). After birth, the gut is colonized by vertical transmission from the mother to the infant and its maturation and composition is shaped by different factors, such as the method of feeding, the mother’s diet or the exposure to medications (e.g. antibiotics) (8–11). The microbiome composition becomes more stable after the first two years of life and is mostly characterized by Firmicutes and Bacteroides (7).

The gut microbiome has been extensively investigated over the last decade to dissect its physiological function and its association with gut and liver diseases. Although many aspects of the gut microbiome are yet to be clarified, it maintains stability of the gut barrier and the maturation of the mucosal associated immunology, and its primary function is the involvement of a large number of metabolic processes (12–15).

## Role of Bile Acids in Gut-Liver Axis

**Figure 1:**
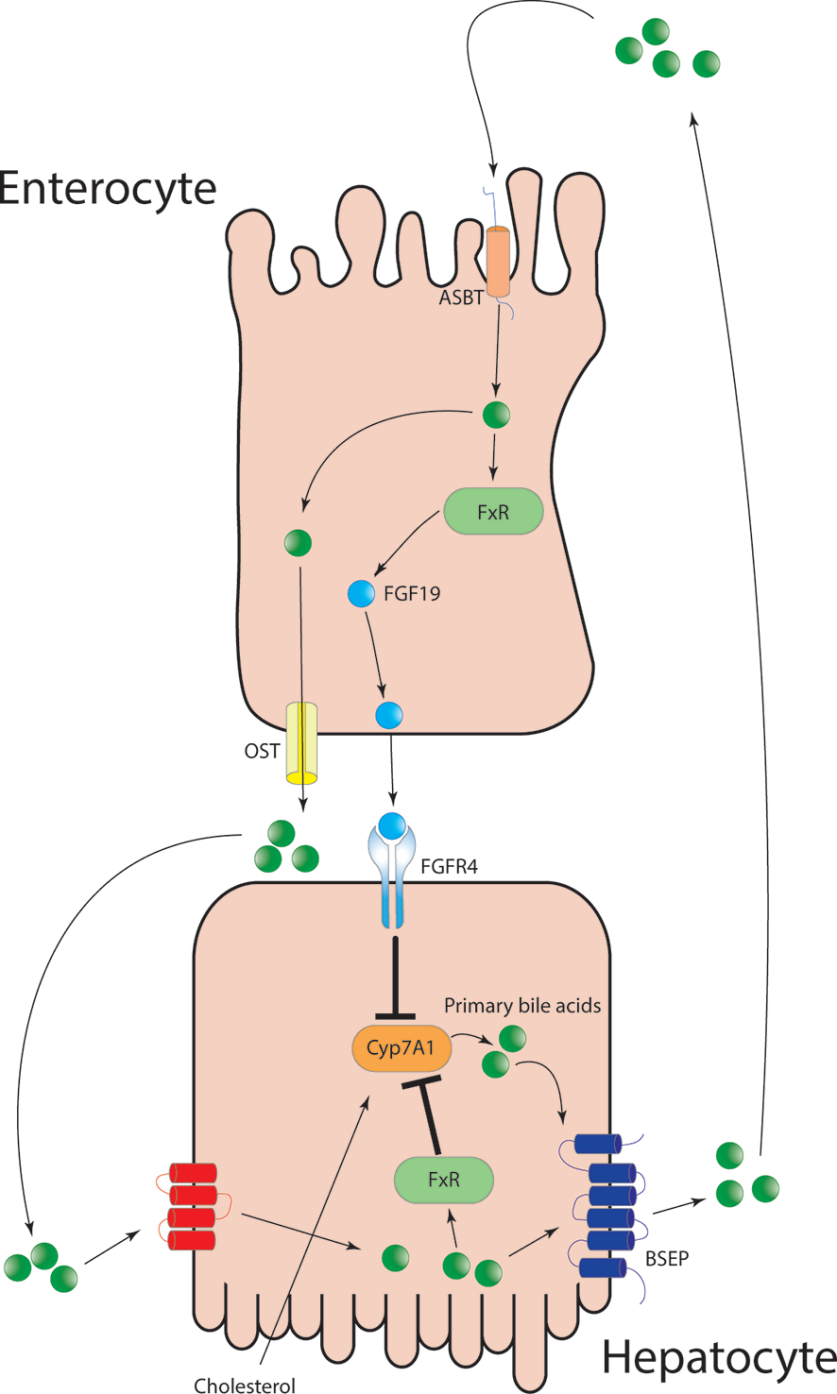
Primary bile acids after being secreted from the hepatocytes into the bile, are reabsorbed in the terminal ileum *via* the apical-sodium dependent bile acid transporter. The increased intracellular concentration of BAs is sensed *via* FXR and lead to the production of fibroblast growth factor 19 and to its secretion into the portal circulation. FGF19 binds FGF4 on hepatocytes surface and lead to the downregulation of Cyp7A1 and in turn inhibiting *de-novo* primary bile salt synthesis.

One metabolic process is the metabolism of the biliary acids (BAs): the primary bile acids, namely the cholic acid and the chenodeoxycholic acid, are synthesized and secreted in the bile by the hepatocytes. The BAs are essential for the digestion of the lipids and the fat-soluble vitamins. The metabolism of the BAs is a paradigmatic example of the interaction between the gut and the liver. Primary BAs recirculate in a highly efficient enterohepatic circulation, and only around 10% of the secreted BAs are newly synthesized by the hepatocytes. In fact, a fraction of BAs are reabsorbed passively, whilst most of them are actively reabsorbed through the apical sodium dependent ileal BA transporter expressed by the enterocytes in the terminal ileum. The bile acids bind farnesoid X receptor (FXR) in the enterocyte, promoting the transcription the fibroblast growth factor (FGF 19) (16). FGF 19 binds FGF4 receptor and Klotho-beta, inhibiting the bile acid synthesis (**Figure 1**). A small proportion of the primary BAs, after reaching the colon, are deconjugated by the microflora into the secondary BAs, lithocholic acid and deoxycholic acid. The former is reabsorbed and after being conjugated in the liver is secreted in the bile (16). An abundance of the deoxycholic-acid affects the microbiota composition and has been related to obesity in non-alcoholic fatty liver disease (17). Thus, new treatment such as obeticholic acid has been shown to be hopeful for NAFLD therapy.

They exert the metabolic effect upon the activation of nuclear receptors (e.g. farnesoid X receptor, FXR) and G-protein coupled bile salt receptor, TGR5. The activation of FXR has been shown to improve glucose tolerance and insulin resistance in murine models (18, 19). Upon their action on FXR, bile acids can improve glucose metabolism after a meal. The stimulation of FXR and in turn the induction of FGF19, reduce the plasma glucose and induce glycogen synthesis (20). The FXR signaling also inhibits the glucose-induced transcription of several genes involved in glycolysis (21). The metabolic effects of bile acids extend to lipid synthesis. FXR-/- mice show increased triglycerides both in liver and serum, alongside cholesterol. FXR activation reduces hepatic lipogenesis, increases the synthesis of apolipoprtein CII and A5 and inhibits ApoA1 and ApoCIII, thereby promoting the reduction of serum triglycerides activating lipoprotein lipase in very low-density lipoprotein (VLDL) (22, 23). Finally, FXR stimulates fatty acid oxidation inducing human peroxisome proliferator-activated receptor α (PPARα) (24).

TGR5 activation increases basal metabolism and in turn promotes energy expenditure. Secondary bile acids activate TGR5 in brown adipose tissue in mice or in muscle in human, increasing the basal energy consumption (25, 26). This metabolic change prevents obesity and reduce insulin resistance in mice (27). The effect on the glucose metabolism includes the induction of the glucagon like peptide 1 secreted from enteroendocrine L cells, an incretin secreted after the meal in order to regulate insulin secretion (28).

Ultimately, the bile acids binding the farnesoid X receptor, in the enterocyte promote the gut vascular barrier integrity, preventing the translocation of pathogens in the portal circulation (29). Agonist of both these receptors have been proposed as treatment in liver diseases. Obeticholic acid an FXR agonist, has been approved in 2016 as an add on treatment for ursodeoxycholic acid non-responders in primary biliary cholangitis. This medication has been shown to be effective in reducing liver fibrosis (NASH fibrosis) in non-cirrhotic patients (30).

### Gut Barrier and Mucosal Immune Response

The gut barrier is a functional unit which prevents bacterial adhesion and controls paracellular trafficking. It is composed, starting from the outer layer, by the gut microbiota, the mucus layer which contains antimicrobial products (such as defensin) and the secretory IgA, the epithelium which is both a physical and immunological barrier and the gut associated lymphoid tissue. A new entity has been recently discovered on the gut defense line, the gut-vascular barrier, known as 4KDa large which prevents the translocation of the bacteria from the gut to the bloodstream (31). Its structure resembles the blood-brain barrier, formed by the endothelial cells linked by tight junctions in close contact with pericytes and fibroblast.

The disruption of the gut barrier alongside an overgrowth of Gram negative bacteria has been linked to multiple liver diseases, as NASH and alcoholic liver disease. Inflammatory mediators such as interferon-gamma or toxic molecules like ethanol can affect the functional integrity of the gut-barrier, causing a “leaky gut” (32, 33). This setting would allow endotoxin, Gram negative bacteria and lipopolysaccharide (LPS) to translocate to the portal system and these inflammatory triggers are postulated to be involved in the pathogenesis of chronic liver diseases.

### Hepatic Immunological Response to Gut Permeability

The gut-barrier prevents the translocation of microbial antigen into the portal circulation. Nonetheless, the small part of microbial associated molecular patterns (MAMPs) (e.g., LPS, peptidoglycan or lipoteichoic acid) which eludes the barrier and reaches the liver parenchyma is detected and destroyed before reaching the systemic circulation (34). This is carried out by Kupffer cells which play a major role in the initial immune response creating a sinusoidal firewall against circulating pathogenetic material and bacterial derivatives from the gastrointestinal tract (35). They are anchored to the sinusoidal surface and their protoplasmic processes can insinuate in the endothelial fenestrae to reach the Disse space.

This strategic position of the liver exposes them to a large number of antigens coming from the portal bloodstream. In physiological settings they promote tolerance, reducing the recruitment and activation of T cells including regulatory T cells (Tregs) which control effector T cells *via* suppression of effector cells proliferation and their cytokine secretions (36, 37). A protective barrier has been shown also on the biliary side and it is represented by the Mucosal Associated Invariant T (MAIT) cells (38). These unconventional T cells express a conserved TCR alpha chain Vα7.2-Jα33 and recognize vitamin B metabolites of microbial origins *via* the MHC-related molecules 1 (39). Intrahepatic MAIT cells have been localized in the portal area around the bile tract. They have been found to interact with the biliary epithelial cells to protect the biliary mucosa (40). Liver retained MAIT cells express chemokine receptors CCR6 and CXCR6 homing them close to the biliary epithelial cells (BECs) which express and secrete CCL20 and CXCL16. Thus, when exposed to bacterial products, MAITs degranulates, releasing IFN-gamma upon MR1 stimulation to protect the biliary epithelium (40–42).

The protective function of the gut and liver barriers and the tolerogenic response of the liver are vital for a harmless cooperation between the liver and the gut. The failure of the gut barrier in preventing the translocation of microbe associated molecular patterns (MAMPs) exposes the liver to an increased volume of immune stimuli. Hepatic Kupffer cells, hepatic sinusoidal endothelial cells (HSECs) and biliary epithelial cells (BECs), recognize MAMPs via pattern recognition receptors (PRRs) or nucleotide-binding oligomerization domain like receptors (NLR) and in turn shape the immune liver milieu toward an inflammatory status causing a liver injury and eventually progression to fibrosis (43–45).

A “leaky gut”, alongside a reduction in LPS tolerance has been theorized as a central player in chronic inflammation in liver diseases (46). A typical example of this paradigm is the primary sclerosing cholangitis (PSC). PSC is characterized by chronic inflammation and fibrotic destruction of the biliary system. It is frequently associated with inflammatory bowel disease, particularly with ulcerative colitis (47). The extent of the gut inflammation is associated with the grade of the intestine permeability and, in turn, with the bacterial-derived endotoxin in the portal circulation (48). We, hereby describe the immunological cross-talk between the gut and the liver, focusing on PSC compared with PBC and checkpoint induced hepatitis and colitis with particular attention on completed therapeutic attempts.

## Checkpoint Inhibitors in Gut-Liver Axis

Checkpoint inhibitors (CPI) are currently used as standard of care treatment for melanoma, non-small cell lung cancer, urothelial malignancy, and prostate cancer. Therapies that target coinhibitory receptors or immune checkpoints inhibitors, CTLA-4, PD1, PDL-1, TIGIT, LAG-3 and TIM3 are currently at the forefront of treatment strategies for cancer. Immune checkpoint inhibitor (ICI) therapy is rapidly developing and is positioned as a crucial treatment within the field of oncology. Although these therapies have improved survival rates of patients carrying various tumors (49, 50), ICIs are also associated with immune-related adverse events and reflect their unique mechanisms of action. These adverse events are known to cause severe colitis and liver injury.

Immune checkpoint blockade with mono-clonal antibodies (mAbs) targeting programmed cell death protein 1 (PD-1) provides long-term clinical benefits to nearly 40% of patients with advanced melanoma (51–54). It is now recognized that ICIs are frequently associated with luminal gastrointestinal side effects such as diarrhea and enterocolitis and hepatic complications such as hepatitis.

## Prototype Disease of Gut-Liver Axis: Primary Sclerosing Cholangitis

PSC, a chronic, progressive biliary disease associated with inflammatory bowel disease (IBD), is one of the best examples of immune mediated liver disease related to gut-liver axis. Currently there is neither curative therapy (55) or treatment to slow the progression of PSC to advanced liver disease (56). Genetic studies have shown that HLA class II alleles associated with ulcerative colitis (UC) are different from those found in patients with PSC and patients with PSC and inflammatory bowel diseases (IBD) (57).

While the pathogenesis of PSC has not been fully elucidated, one hypothesis involves the migration of gut mucosal memory lymphocytes to the liver which then causes focal inflammation and fibrosis within the large and/or small bile ducts (58, 59). Current available standard medical therapy only includes ursodeoxycholic acid (UDCA) which does not benefit the overall survival. Liver transplantation remains the only curative therapy in PSC (60, 61). 60-80% of PSC patients have coexistent IBD (62). The phenotype of IBD in PSC has particular characteristics, such as involvement of the entire colon with right-sided dominance, ileal inflammation, and relative rectal sparing. It has been suggested that the IBD associated with PSC is its own distinct entity separate from Ulcerative colitis (UC) or Crohn’s disease (CD) alone (63).

Sufficient epithelial barrier function as well as innate and adaptive immune regulation is required for a lifelong balanced response to dietary antigens as well as for commensal and pathogenic organisms which can infect the small and large intestine. If the immune regulation fails because of changes in environment and lifestyle, the accumulation of common genetic susceptibility variants, chronic intestinal inflammation can arise. Inflammatory bowel disease (IBD) encompasses Crohn’s disease (CD) and ulcerative colitis (UC). IBD is characterized by chronic relapsing disease activity of acute flares and intervals of remission (64, 65).

One hypothesis to explain the co-occurrence of IBD and PSC due to the interaction between the gut and the liver is known as ‘the aberrant gut homing lymphocyte hypothesis’ (58). This hypothesis describes an aberrant expression of gut homing molecules such as CCL25 and MadCAM-1 in PSC liver, leading to homing of CCR9 and α4β7 expressing gut-primed CD8 memory T-cells into the liver. In patients with PSC-IBD, there is a recurrence of PSC following transplantation if small bowel or colon is still intact (66). This would explain the mechanism for T cell trafficking between the gut towards the liver supporting the important mechanism of liver-gut immune axis in these diseases (67). Current standard of care treatment for IBD not only includes immunosuppressive medication and biologic therapies such as anti-TNF therapies (infliximab), α4β7 blockade (Vedolizumab) is now routinely used for IBD patients to prevent mucosal T cell homing (68). Sequencing of the TCRβ repertoire data demonstrated that memory T cells of common clonal origin were detected in paired gut and liver samples of patients with PSC-IBD. This suggests that memory T cells driven by shared antigens are present in the gut and liver of PSC-IBD patients (69). Therefore, therapeutical targeting memory T cell recruitment in PSC-IBD with Vedolizumab is a beneficial therapy.

‘PSC microbiota’ or the ‘leaky gut’ theory implies that the pathogenic gut microbiota crosses through the inflamed gut wall into the portal circulation, and then into the biliary tree, leading to PSC (70). We currently have fecal microbiome treatment for IBD patients and this will be extended to PSC patients in the near future.

## Primary Biliary Cholangitis: Is There a Gut-Liver Link?

Primary biliary cholangitis (PBC) is a chronic autoimmune cholestatic liver disease. The immune damage targets the small and medium intrahepatic biliary ducts, causing a progressive damage which eventually leads to fibrosis and cirrhosis (71). The PBC pathogenesis is still enigmatic and the current hypothesis is that environmental factors (e.g. infections) can start the process in individuals with a genetic predisposition (72).

A damage in the gut barrier in PBC has been hypothesized as a pathogenetic factor. The higher permeability of the small intestine shown in PBC (73, 74), might be at the basis of the increased LPS levels observed in PBC patients compared with healthy controls (75). Intriguingly, long-standing exposure to bacterial antigens causes the development of PBC-like histological features in BALB/c mice (76). Altogether these evidences suggest a failure in gut barrier in PBC patients.

The interest around this pathogenic angle has recently increased as well as the effort to investigate the microbiome in PBC (77–79). Microbiome alpha diversity is reduced in PBC and it shows a distinct beta diversity compared with healthy controls due to the increased of the genera Streptococcus, Lactobacillus, and Bifidobacterium (80). The interplay between bile acids metabolism and microbiome in PBC has been nicely elucidated by Chen and colleagues. They showed how the BA composition in serum and feces significantly differ in UDCA-naïve PBC patients compared with healthy controls. PBC BA pool was associated with a decreased conversion of conjugated and unconjugated, and primary to secondary BAs. These results suggest an impaired microbial BA metabolism. This effect appears to be related with the severity of the disease, as the BA abnormality correlates positively with the disease stage (77). The first line of treatment in PBC is ursodeoxicholic acid (UDCA) which seems, to have a role in restoring the gut microbiome in PBC patients (80).

## Inflammatory Bowel Disease and PSC

**Figure 2:**
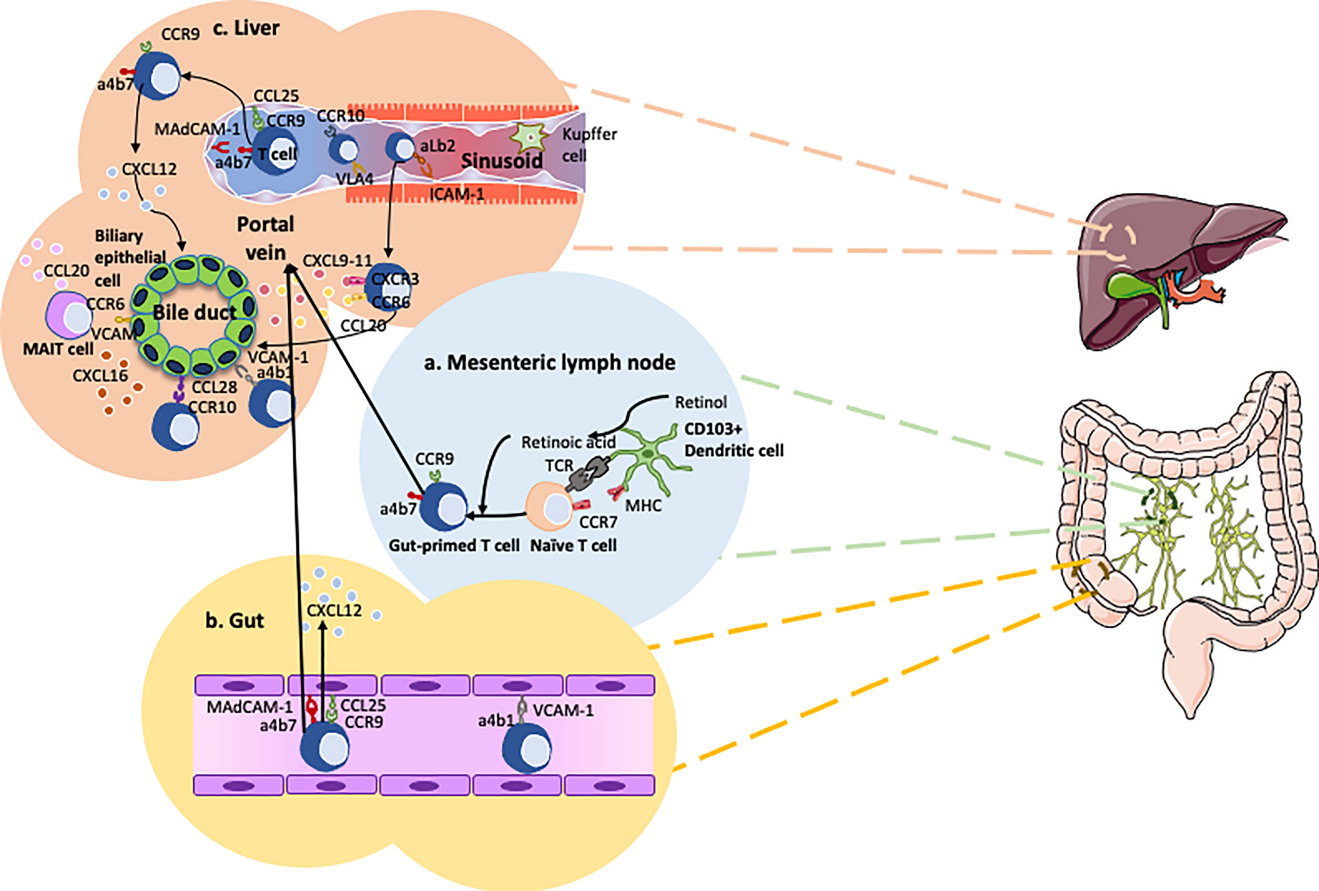
Overview of the gut-liver axis homing of cells in primary sclerosing cholangitis (PSC). **(A)** Dendritic cells recognize pathogens penetrating the mucosal barrier and subsequently migrate to the mesenteric lymph node to present their antigens to naïve T cells (82). These naive T cells are imprinted with the gut-homing integrin α_4_β_7_ and chemokine receptor CCR9 *via* the transformation of retinol into retinoic acid. **(B)** The gut-primed T cells recirculate into the gut bind onto the endothelium of the blood vessels *via* interactions between MAdCAM-1 and α_4_β_7_, CCL25 and CCR9. **(C)** CCR10 expressing Tregs and gut-primed memory T cells expressing α_4_β_7_ and CCR9 migrate from the gut to the liver *via* the portal vein. These concepts are applied in PSC to develop new therapies such as Vedolizumab. Chemokine CXCL12 is thought to play a role in maintaining CCR9+ lymphocytes around the bile ducts (83).

Chemokines and chemokine receptors crosstalk are essential in gut-liver immune traffic. Lymphocyte recruitment to the gut from the circulation involves a range of chemokines and adhesion molecules. Chemokine CCL25 aids in the trans-endothelial migration of gut-homing T-cells, so enhanced expression will promote infiltration of T cells. Expression of CCL25 is normally confined to the epithelium and mucosal vessels in the small intestine (81), where it interacts with gut-homing B and T cells expressing its receptor, CCR9 (**Figure 2**). In the inflamed liver there have been reporting’s of strong expression of CCL25 on the hepatic vessels (47). Although CCL25 is largely absent from the non-inflamed human colon, expression is markedly upregulated in colitis and correlates with inflammatory activity (84). CCL25 expression in the colon is associated with high frequencies of CCR9^+^ tissue-infiltrating effector T-cells in patients with colitis. Thus, CCR9 and CCL25 interactions drive the recruitment of mucosal effector cells in the gut as well as the liver in patients with ulcerative colitis (84). Once an antigen is recognized by gut dendritic cells, they imprint naïve T cells with gut specific chemokine receptor CCR9 and integrin, α4β7 (85). These primed T cells subsequently migrate back to liver (47, 83, 86). Deamination of methylamine by vascular adhesion protein-1 (VAP-1) - semicarbazide sensitive amine oxidase found in the human liver – induces the expression of function MAsCAM-1 in hepatic endothelial cells and intact human liver tissue *ex-vivo*. This is associated with increased adhesion of lymphocytes from patients with PSC to hepatic vessels (87). It has been reported that there is an aberrant expression of the gut-specific molecules MAdCAM-1 and CCL25 in human PSC livers (47, 59, 88). This correlates with α4β7^+^CCR9^+^effector memory T-cells which constitute approximately 20% of the liver-infiltrating lymphocytes in PSC patients (47). This would explain the recurrence of PSC after liver transplantation in patients with intact inflamed colon.

Due to MAdCAM-1 expression by portal endothelium coupled with the presence of chemokine CCL28 and integrin α1β7 around the bile ducts, the inflammatory trigger in PSC originates in the portal area e.g. in the bile ducts, thereby provoking cholangitis and not hepatitis (89). Krijger et al. demonstrated the expression of CCL28 in PSC liver (89). CCL28 is also upregulated in the epithelium of the colon during inflammation, subsequently attracting T and B cells expressing CCR10 (90). Studies using flow-based adhesion assays demonstrate CCL28 is able to trigger α4β7-dependent lymphocyte arrest (83). In addition, VCAM-1 expression by cholangiocytes promotes the survival of intrahepatic α4β7 expressing effector T lymphocytes subsequently contributing to the persistent inflammation (91).

To translate our knowledge of discovery science to clinics, several compounds have been developed to tackle and block the migration of gut-homing mucosal lymphocytes to treat patients with IBD and PSC. These compounds block α4β7 (vedolizumab, abrilumab), integrin β7 (etrolizumab), and MAdCAM-1 (ontamalimab). Although studies have focused predominantly on the treatment of IBD with these compounds, their potential to block trafficking to the liver suggests they could also be a future treatment option for PSC-IBD and also for inflamed bowel even after liver transplantation (92, 93).

## Translating Immunology to Therapy: Vedolizumab in PSC and IBD

Vedolizumab is a humanized monoclonal antibody that binds specifically to the α_4_β_7_ integrin, a mediator of gastrointestinal inflammation (94, 95). Blocking α_4_β_7_ on memory T-helper lymphocytes by vedolizumab inhibits the adhesion of cells to MAdCAM-1 which is expressed on inflamed portal vein endothelium in patients with IBD and PSC. The inhibition of this interaction prevents the transmigration of gut-homing memory T cells across the vascular endothelium leading to reduction in inflammation (96, 97). Safety and efficacy of vedolizumab has been demonstrated in large-scale GEMINI clinical trials in both Crohn’s disease (CD) and ulcerative colitis (UC). Clinical remission rates of 39% and 42% was achieved with vedolizumab in both CD and UK respectively, and this effect was maintained for a year (98, 99). This led to the approval of vedolizumab by the UK Food and Drug Administration (FDA) and European Medical Agency (EMA) in 2014 to treat CD and UC. Studies since its approval have proven its efficacy [e.g., the VERSIFY study (100)] and the efficacy of early versus late use in CD [LOVE-CD trial (101)]. Cohort studies have also demonstrated improved disease control, corticosteroid sparing and a decline in concomitant drugs, with a significant proportion of patients remaining on vedolizumab by the end of the observation period (102).

Several studies have suggested that the gut-homing pathways that vedolizumab targets is implicated in the pathophysiology of PSC (47, 59, 103). Therefore, vedolizumab may play a role in reducing lymphocyte infiltration in PSC and thereby in reducing hepatic and biliary inflammation. However, there have been varied findings on the efficacy of vedolizumab in PSC. Researchers have shown that vedolizumab therapy did not significantly improve markers of biliary inflammation (104, 105), however, vedolizumab can induce clinical remission in rheumatologic extraintestinal manifestations of IBD (106). Patients with more aggressive disease, such as the presence of cirrhosis and potentially those with a raised ALP at baseline, were more likely to respond (107).

## Manipulating Gut Microbiome With Vancomycin in PSC

The microbiota plays an important role in the pathogenesis and the progression of PSC. This concept was applied recently to treat patients with PSC. Antibiotics, such as vancomycin, was utilized in clinical trials to modify intestinal microbiota flora. Trial data demonstrated that vancomycin could improve liver biochemistry in PSC patients (108). This evidence suggested that disease progression in PSC can be modified by antibiotic therapies.

Vancomycin is a glycopeptide antibiotic with bactericidal activity against Gram positive bacteria (109) which includes various *Clostridium* spp. known to be primarily involved with the dihydroxylation of primary bile acids, into the secondary bile acids present in the distal small intestine and colon (110, 111). Secondary bile acids are highly hydrophobic and toxic, and increased concentrations in the liver have been linked to inflammation, cholestasis and carcinogenesis (112). Therefore, vancomycin could influence bile acid metabolism. A meta-analysis study by Shah et al. exploring the effects of antibiotics vancomycin and metronidazole in PSC patients demonstrated that overall antibiotic therapy significantly improved primary outcome measure alkaline phosphatase enzyme level. The use of vancomycin in patients with PSC have not only biochemical benefits but also cholangiography and histological improvements to PSC and decreased intestinal inflammation of the IBD on colonic biopsies (113). Multiple studies have reported similar results in both adults and children with PSC (108, 114–116) supporting that modification of intestinal microbiota has beneficial effects by changing gut-liver immune dynamics.

## Potential Role of Regulatory T Cell Therapy in PSC

Tregs are crucial to maintain control of the magnitude of immune responses to self-antigens and to limit tissue damage caused by immune activation in response to these antigens (117). The immune-regulatory function is impaired in autoimmune or inflammatory diseases. Thus, Treg therapy in PSC is worth exploring as a potentially curative therapy. IL-2 infusion therapy to expand Tregs *in vivo* has been investigated in mouse models of cholangitis reporting a 5-fold increase in hepatic Tregs, and cells are localized around the inflamed portal tracts. The increase in Tregs subsequently resulted in reduction of pro-inflammatory IL-17 cytokines and increase in anti-inflammatory IL-10 production by hepatic lymphocytes. Low dose IL-2 infusion therapies have been carried out in human autoimmune diseases (118) and a majority of findings resulted in a decrease in disease severity. Although this has not yet been trialed in autoimmune liver diseases, the current mouse and human data suggest IL-2 infusion alone may not be enough to decrease the disease severity but should be considered in combination with Treg infusion therapies.

Thus, vendolizumab, vanomycin and Treg therapy are the current in-trial strategies with described benefits in aspects of the gut-liver injury related to PSC and IBD. However, further trials are needed to prove their beneficial effects in a high percentage of patients. However, not all patients respond to a particular treatment and therefore it is important that we have a variety of treatments to have the best possible chance to help every patient. Therefore, it is also important that we continue to explore new therapies as well as progressing those that are currently looking promising in the trail phases.

## Immune Mediated Gut-Liver Injury: Checkpoint Inhibitor Induced Colitis and Hepatitis

### Checkpoint Inhibitors Induced Hepatitis

It is not uncommon that checkpoint inhibitors lead to untoward immune mediated injury including hepatitis and colitis. In a study looking at 414 patients undergoing treatment with CPI, 28 (6.8%) were diagnosed with immune related hepatitis (119). The rate of severe acute hepatitis was similar in patients treated with anti-CTLA-4 and patients treated with anti-PD1/PD-L1. Immune related hepatitis is one of the highest occurring adverse event associated with immunotherapy, especially when anti-CTLA-4 and anti-PD1/PD-L1 are prescribed as a combination therapy (120, 121). Immunological changes resulting from CPI drugs may contribute to the immune related hepatitis. Peripheral CD8 T cells in CPI induced hepatitis showed elevated activation/cytotoxicity and is associated with peripheral monocyte activation (122). CD8 T cells/macrophage were also shown to aggregate in CPI induced hepatitis patients shown using immunohistochemistry staining.

### Check Point Inhibitors Induced Colitis

**Figure 3:**
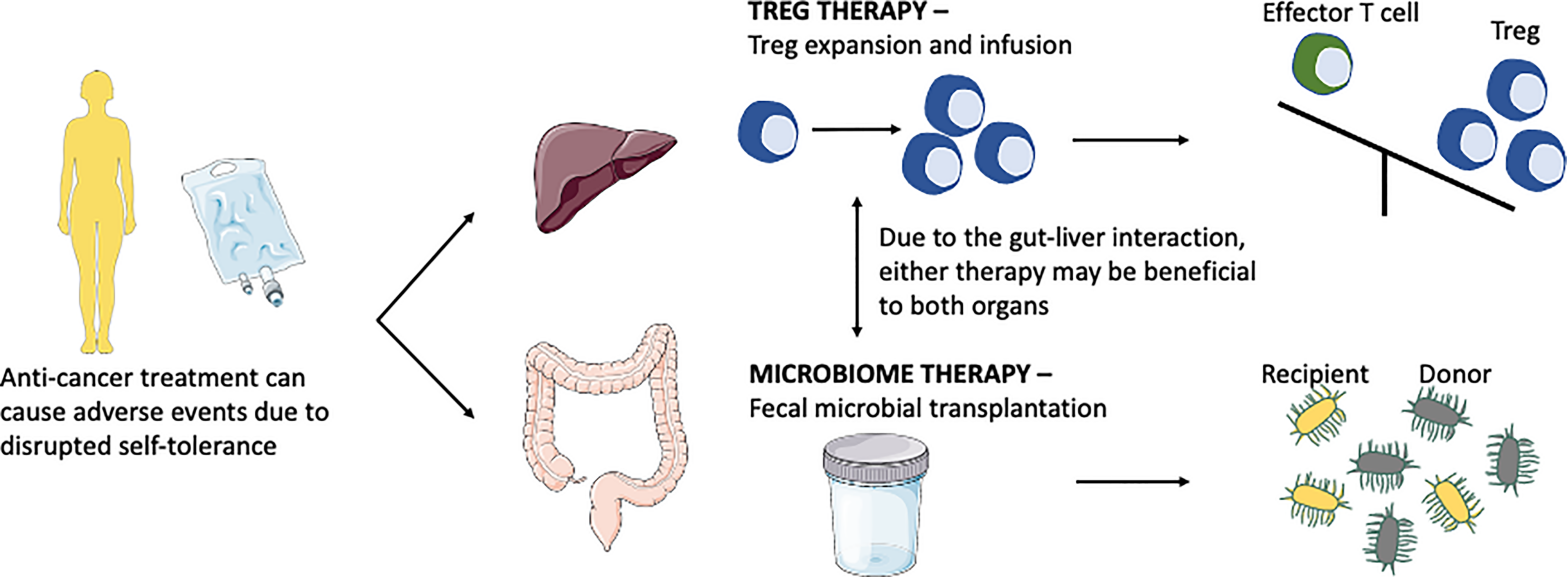
Anti-cancer treatment disrupts self-tolerance leading to colitis and hepatitis, current research is focusing on microbiome therapy and Treg therapy to combat these diseases. Treg therapy aims to switch the balance from the effector cells arm to the Treg arm. Microbiome therapy *via* fecal microbial transplantation aims to modulate the microbiome by direct interaction or competition leading to host immunity modulation.

Check point inhibitors can lead to colitis as an adverse effect. Recent summary work on colitis suggested that most cancer patients were treated with CTLA-4 inhibitors, followed by PD-1 inhibitors, or combination therapy. A study on a total of 226 patients with CPI-induced colitis suggested that these patients were treated with steroids (oral or intravenous). Because CPI induced injury is an immune mediated event, it responds to immunosuppressive therapy and biologic treatment which targets the immune pathway. Of these, 61% responded to steroids alone whilst 47% required further treatment with anti-TNF, infliximab. 94% of patients treated with infliximab had resolution of colitis. 8 patients were treated with vedolizumab after steroid failure and all of these patients had resolution (123). Thus, global immunosuppression with steroid, manipulating TNF and blocking α_4_β_7_ on memory T-helper lymphocytes are current approach of treatment is these cases. Excitingly, new research focuses on understanding the role of modifying microbiome in the gut and also to enhance the regulatory arm of the immune system to treat CPI induced colitis and hepatitis (**Figure 3**).

### Potential of Fecal Microbiome Therapy CPI-Induced Colitis and Hepatitis

Microbiome-targeted therapies manipulate the gut micro flora and can be considered in several categories, namely probiotics, synbiotic, antibiotics and fecal microbiome therapy **(**FMT). Probiotics are defined as a culture of living microorganisms which could have health benefits for the human if consumed in adequate amounts (124). Synbiotics are a combination of probiotics and prebiotics, in which prebiotics are composed of fermentable dietary fibers that stimulate the growth and survival of probiotics (124). Vancomycin is a typical example of antibiotic which change gut flora.

FMT is a procedure in which stool is collected from a healthy donor and transferred to a patient, *via* a range of delivery routes including colonoscopy, nasogastric tube, and enema (125). The gut microbiome is a major regulator of responses to anti-PD-1 (126–128). In mice, composition of the gut microbiome modulates therapeutic activity and anti-PD-1 and anti-programmed death-ligand 1(PD-L1), and administration of FMT promotes anti-PD-1 efficacy in melanoma bearing mice (129–131). A single FMT administered colonoscopically together with PD-1 blockade successfully colonized the gut of patients and reprogrammed the tumor microenvironment to overcome primary resistance to anti-PD-1 in a subset of patients with advanced melanoma (132).

The STOP-COLITIS trial (lead by University of Birmingham) is currently ongoing for FMT therapy in non-CPI colitis and the data is encouraging. A recent study applying FMT to treat 2 cases of CPI-colitis demonstrated that it is effective in a subset of patients. Although both patients received stool from the same donor, they experienced contrasting treatment responses. One patient had a high level of activation (as measured by co-expression of HLA-DR and CD38) on CD4, CD8 and MAIT cell subsets as well as high Ki-67 and low expression of Bcl-2 before FMT treatment. Post FMT, there was reduced expression of HLA-DR and CD38 and lower expression of Ki-67 with high/homeostatic levels of Bcl-2 and an increased proportion of Tregs (133). Thus, actual mechanism and predictor of FMT treatment still requires further investigations.

In the context of liver disease and FMT application, the gut microbiota has been implicated as a central factor in the pathophysiology of PSC. Commensal microbiota and its metabolites protect against biliary injury (134). PSC patients not only display a characteristic microbial signature but also have inflammatory bowel disease, thereby suggesting that manipulation of the gut microbiota could potentially influence the disease process in PSC. Thus, FMT therapy to treat gut-liver disease such as PSC is a new and upcoming therapeutic option. One pilot study conducted by Allegretti et al. demonstrated the safety of FMT in 10 patients with PSC, they also suggested that the subsequent increases in bacterial diversity and engraftment may correlate with an improvement in Alkaline phosphatase enzyme among patients with PSC (135).

## Future Directions

Understanding the immunological link between the gut and the liver is essential for the success of curative therapies to treat related diseases. Current treatment for IBD patients is hampered by side effects such as myelotoxicity, sepsis, or reactivation of opportunistic infections. To date, there is no treatment in PSC. In addition, only global immune suppression with steroids is generally applied for checkpoint induced hepatitis and colitis in Oncology arena. Thus, dissecting immune pathway is crucial in targeted and organ-specific personalized therapy based on pathogenic mechanisms. New technology including single cell genomic, proteomic, metabolomic, microbiome (Multi-OMICs) profile of patients will guide us to apply treatment as a stratified medicine approach with reduced side effects. FMT therapy currently seems to be the most promising but future studies with substaintial patient numbers are needed to confirm that this is a beneficial treatment. For this to be an effective and curative therapy, exploring the tissue resident cell gene, protein and functional signatures and understanding their microenvironment is an absolute requisite. Application of modern techniques such as single cell RNA sequencing, adaptive cells clonotype and spatial gene signature expression of gut-liver immune mediated diseases would guide us to achieve a personalized medicine approach in future.

## Author Contributions

AB conceptualized, collected the data, wrote, and edited the manuscript as well as creating the illustrations. VR conceptualized wrote, and edited the manuscript. DO-B contributed to the writing of the manuscript. YO conceptualized and edited the manuscript and supervised the work. All authors contributed to the article and approved the submitted version.

## Funding

Transbioline Innovative Medicine Initiative, Sir Jules Thorn Biomedical Research Award, Medical Research Foundation, European Association of Study of Liver Disease Juan Rodes Fellowship, NIHR BRC Birmingham, GSK, Queen Elizabeth Hospital Birmingham Charity.

## Conflict of Interest

The authors declare that the research was conducted in the absence of any commercial or financial relationships that could be construed as a potential conflict of interest.

## Publisher’s Note

All claims expressed in this article are solely those of the authors and do not necessarily represent those of their affiliated organizations, or those of the publisher, the editors and the reviewers. Any product that may be evaluated in this article, or claim that may be made by its manufacturer, is not guaranteed or endorsed by the publisher.
